# Exosomes secreted by chemoresistant ovarian cancer cells promote angiogenesis

**DOI:** 10.1186/s13048-020-00758-w

**Published:** 2021-01-07

**Authors:** Zhang Li, Wang Yan-qing, Yang Xiao, Liu Shi-yi, Yuan Meng-qin, Xian Shu, Yang Dong-yong, Zheng Ya-jing, Cheng Yan-xiang

**Affiliations:** 1 Department of Obstetrics and Gynecology, Renmin Hospital of Wuhan University, Wuhan, 430060 China; 2 Department of Anesthesiology, Renmin Hospital of Wuhan University, Wuhan, 430060 China

**Keywords:** Ovarian cancer, Drug resistance, Exosomes, Angiogenesis, miR-130a

## Abstract

**Background:**

Ovarian cancer (OC) has the highest mortality rate in gynecologic tumors. Despite decades of continuous efforts, the survival rate of patients has not improved significantly, mostly due to drug resistance. Exosomes are hot topics in recent years. Cells can affect the biological behaviors of other cells by transferring exosomes. So far, numerous researchers have found that tumor cells can secrete exosomes which play a important role in the development of tumors. Solid tumors can promote angiogenesis. When drug resistance occurs, it seems that more blood vessels form. We suppose that exosomes derived from chemoresistant OC cells can also promote angiogenesis.

**Results:**

We investigate whether exosomes secreted by chemoresistant SKOV3-DDP cells (SKOV3-DDP-exo) and sensitive SKOV3 cells (SKOV3-exo) influence angiogenesis. After exosomes were extracted, exosomes were co-cultured with HUVECs. We found that SKOV3-DDP-exo and SKOV3-exo are absorbed by endothelial cells and promote the proliferation, migration, invasion and tube formation of endothelial cells. Moreover, SKOV3-DDP-exo is more powerful in angiogenesis, suggesting that parts of the components of SKOV3-DDP-exo are significantly radical. We also found that miR-130a was highly expressed in drug-resistant OC cells. Also, we found that miR-130a in SKOV3-DDP-exo is higher than SKOV3-exo. Therefore, we suggest that miR-130a in exosomes is the main cause of chemoresistant OC cells promoting angiogenesis.

## Introduction

Ovarian cancer (OC) ranks first in the mortality rate of gynecologic cancers. Because early symptoms are latent, most patients are found to be advanced. Standard treatment for advanced OC is cytoreductive surgery combined with cisplatin-based chemotherapy. Initially, the response rate of OC to cisplatin reaches 80% and chemotherapy has significantly inhibited tumor growth, however, due to drug resistance, up to 70% of patients will eventually relapse, the 5-year survival rate still remains at approximately 30% [1, 2]. Although many studies have been done to solve the problem, it is still far from enough to address tumor progression after drug resistance.

Tumor progression not just depends on self-characteristics but also the interaction between tumor cells and stroma cells in the tumor microenvironment [3]. The tumor microenvironment consists of various types of cells, including endothelial cells, immune cells, fibroblasts and mesenchymal stem cells, etc [4, 5] Recent studies have found that exosomes play important role in cell-cell communication. Exosomes are vesicles with a diameter of 30–150 nm [6], rich in lipids, proteins, RNA and DNA. Various cells such as epithelial cells [7], neurons [8], T cells [9], B cells [10] can secrete exosomes. By fusing with cell membranes, exosomes are released into the extracellular microenvironment, then participate in cell-cell communication [11, 12]. Studies have found that exosomes mediate signal transduction between adjacent or distant cells and present in various body fluids such as human blood and urine [13, 14]. Tumor-derived exosomes empower to regulate tumor cell biological behaviors such as proliferation [5], immunomodulation [15], metastasis [16, 17], drug resistance [18], etc. Moreover, Riches et al. has revealed that breast cancer cells release 50% more exosomes than human mammary epithelial cells [19], which is consistent with OC, the number of exosomes isolated from the serum of patients is 5 times higher than that of patients with benign disease [20]. In addition, Safaei et al. found that chemoresistant OC cells released 2.6 times more exosomes than parental cells previously [21]. However, these mechanisms of these exosomes in tumor progression is unclear.

The rapid growth of solid tumors cause the occurrence of new blood vessels. It has been found that exosomes in tumor microenvironment can enhance angiogenesis. For example, Bai et al. has discovered that exosomes derived from gastric cancer cells contribute greatly to angiogenesis though miR-135b targeting forkhead box O1 in endothelial cells [22]. Lu et al. unearth that exosomes secreted by nasopharyngeal carcinoma cells can be absorbed by vascular endothelial cells to inhibit angiogenesis, thereby inhibiting the metastasis of nasopharyngeal carcinoma [23]. However, there is no clue that exosomes secreted by drug resistant OC cells contribute to angiogenesis. We suggest that exosomes secreted by drug sensitive and resistant OC cells influence angiogenesis, and suppose that angiogenesis provides more nutrients which promotes the synthesis and secretion of more exosomes in OC cells, especially in chemoresistant cells.

## Materials and methods

### Cell culture

The human cisplatin-sensitive SKOV-3 cells, resistant SKOV3-DDP cells, and normal ovarian epithelial cells IOSE80 were purchased from Cell Bank of Type Culture Collection of the Chinese Academy of Sciences (Shanghai Institute of Cell Biology) and cultured in DMEM (Gibco) and RPMI 1640 supplemented with 10% FBS (Gibco), respectively. The human umbilical vein endothelial cells (HUVECs) were purchased from Sciencell Company (USA) and cultured in Eagle’s minimum essential medium (Wisent, China) supplemented with 10% FBS (Gibco). All cell lines were cultured in a humidified incubator containing 5% CO_2_ at 37 °C.

### Isolation of exosomes

The commercially available GET™ Exosome Isolation Kit (*GenExosome Technologies Inc., USA*) was employed as described by the vender for the isolation of exosomes in the cultured supernatant according to the manufacturer’s instructions. EVs were stored at 4 °C and used within 72 h or were frozen at − 80 °C.

### Transmission electron microscopy

50 μL of exosome suspension was added to the loaded copper mesh, and the excess liquid on the copper mesh was blotted dry from the side with a filter paper. After 1 min, a phosphotungstic acid solution (pH=7.0) was added dropwise to the copper mesh at room temperature for 10 min. And the exosomes were observed under a transmission electron microscope and photographed.

### Nanoparticle tracking analysis

The ZetaView PMX 110 (Particle Metrix, Meerbusch, Germany) was used for real-time characterization of the vesicles. A 488 nm laser was chosen for each sample. Five 60 s videos were recorded and analyzed by Software ZetaView 8.04.02 SP2, and the final results represent the mean and mode.

### Western blot

The isolated exosomes were collected and the protein concentration determined by using the BCA protein assay (Pierce, Waltham, MA). Protein samples were subjected to electrophoresis on polyacrylamide gels (Bio-Rad, Hercules, CA) and then transferred to polyvinylidene fluoride membranes (Bio-Rad). Subsequently, membranes were blocked, rinsed, and incubated with primary antibodies against HSP70 (1:1000), CD9 (1:500) and CD63 (1:500), which were used as exosomal markers (abcam, England). After overnight incubation at 4 °C, membranes were washed and incubated with their corresponding secondary antibody conjugated with horseradish peroxidase. Protein bands were detected with an enhanced chemiluminescence detection kit (GE Healthcare, Piscataway, NJ) and visualized through h a chemiDocxrs imaging system and analysis software (Bio-Rad, San Francisco, CA).

### Uptaking of exosomes

To detect the uptake of exosomes by HUVECs, purified exosomes were cocltured with HUVECs. Briefly, 1 μL PKH26 dye (a red fluorescent labeling kit, Sigma, USA) was added to 500 μL exosome solution which was diluted to 10^9^ /mL, incubated at room temperature for 5 min. Then, exosomes were re-extracted and isolated as mentioned above. The labeled exosomes were incubated with HUVECs which were labeled by Hoechst 33342 at room temperature for 24 h. HUVECs were subsequently washed with PBS and fixed in 4% paraformaldehyde for 15 min. Fixed cells were washed with PBS, and cell nucleus were stained with DAPI. Eventually, the fluorescence uptake was monitored using confocal microscopy (Leica TCS STED CW).

### Cell viability experiment

Cell viability was tested through cell counting kit-8 (CCK-8, Beyotime Biotechnology, Shanghai, China). First, HUVECs were seeded in 96-well plate with 5 × 10^4^ cells/well and 100 μL exosome solution collected from cells SKOV-3 (SKOV3-exo) or SKOV3-DDP (SKOV3-DDP-exo) or PBS were added for 48 h. Then, CCK-8 solution was added to the medium, incubated for 2 h in a humidified incubator containing 5% CO_2_ at 37 °C. 450 nm was used to test absorbance through multifunctional microplate reader spectraMaxM5 (Molecular Devices, San Jose, CA).

### Scratch wound experiment

Migration was evaluated by scratching a confluent layer of HUVECs in a 6-well plate (Corning Costar, Corning, NY) using a P200 pipette tip. After PBS washing, 500 μL SKOV3-exo or SKOV3-DDP-exo or PBS were added for 48 h, and the plate was incubated in a humidified incubator containing 5% CO_2_ at 37 °C. After which the reduction in the wound area was determined using Image-Pro Plus software (Media Cybernetics, Rockville, MD, USA).

### Invasion experiment

For invasion experiment, 5 × 10^4^ HUVECs were plated in a 6-well transwell plates (Corning Costar, Corning, NY) and incubated with 500 μL exosome solution collected from cells SKOV-3 or SKOV3-DDP or PBS. Briefly, cells suspended in serum-free culture medium were added to the top chamber of transwell filters and allowed to migrate through the filter. The lower chamber was full of culture medium containing 20% FBS, added with 500 μL exosome solution collected from cells SKOV-3 or SKOV3-DDP or PBS, then incubated for 48 h in a humidified incubator containing 5% CO_2_ at 37 °C. The cells in the upper chamber were removed, and the cells across the membrane were fixed with 4% formaldehyde, stained with 1% crystal violet dissolved in methanol and the number of cells was counted by inverted microscope.

### Tube formation experiment

To examine the tube formation of the HUVECs that had incorporated exosomes, 2 × 10^4^ cells were seeded into 24-well plates coated with Matrigel (Invitrogen, USA). HUVECs were serum-starved in culture medium for 48 h. Next, the cells were incubated with 200 μL SKOV3-exo or SKOV3-DDP-exo or PBS for another 48 h in a humidified incubator containing 5% CO_2_ at 37 °C. The dynamics of HUVECs behavior was monitored using inverted microscope.

### Quantitative real time PCR

Total miRNAs from cultured cells were extracted with Trizol Reagent (Invitrogen™) according to the manufacturer’s instruction. cDNA was synthesized using the PrimeScript™RT reagent Kit (TaKaRa). Quantitative real-time PCR (qRT-PCR) was performed on a CFX-1000 real-time PCR system (Bio-Rad). The relative miRNA expression levels were calculated by the 2^−△△Ct^ method and normalized to U6. The specific primer sequences used were as following:

**Table:** 

U6:	Forward	CTCGCTTCGGCAGCACAT
	Reverse	AACGCTTCACGAATTTGCGT
hsa-miR-130a:	Forward	CCAGTGCAATGTTAAAAGGGC
	Reverse	CTCAACTGGTGTCGTGGAGTC.

### Statistics analysis

Data analysis was performed using the Origin software version 8.5. Each experiment was carried out in triplicate at least and all results were presented as mean ± s.d. A value of *p* < 0.05 was considered significant.

## Results

### Isolation and quantification of exosomes

**Fig. 1:**
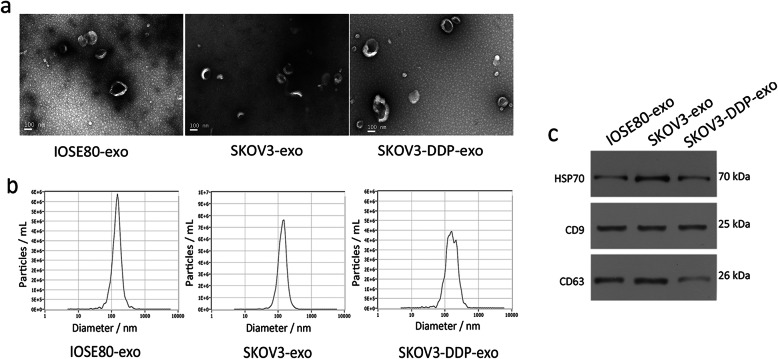
Characterization of exosomes derived from cell lines IOSE80, SKOV3, and SKOV3-DDP. **a** Transmission electron microscopy images of IOSE80-exo, SKOV3-exo, and SKOV3-DDP-exo. Scale bars = 100 nm. **b** Size distribution (nm) of IOSE80-exo, SKOV3-exo, and SKOV3-DDP-exo were determined by Software ZetaView. **c** Western blot analysis of surface markers HSP70, CD9, and CD63 in IOSE80-exo, SKOV3-exo, and SKOV3-DDP-exo

The vesicles are extracted from diverse cultured medium using the GET™ Exosome Isolation Kit after 24 h as mentioned before. The diameter of the vesicles is approximately 90–150 nm and with a double membrane structure observed by transmission electron microscopy and ZetaView (Fig. 1a and b). Moreover, the exosome-specific proteins CD9, CD63 and HSP70 are detected by western blot, as shown in the Fig. 1c. The above confirms that the vesicles we extracted from the cells are exosomes. We observe that drug sensitive SKOV3 cells and resistant SKOV3-DDP cells release more exosomes compared to normal ovarian epithelial cells IOSE80. Besides, it is surprised that SKOV3-DDP cells release more exosomes than SKOV-3 cells do.

### Exosomes secreted by SKOV3-DDP or SKOV3 cells enhance HUVECs proliferation, migration and tube formation

**Fig. 2:**
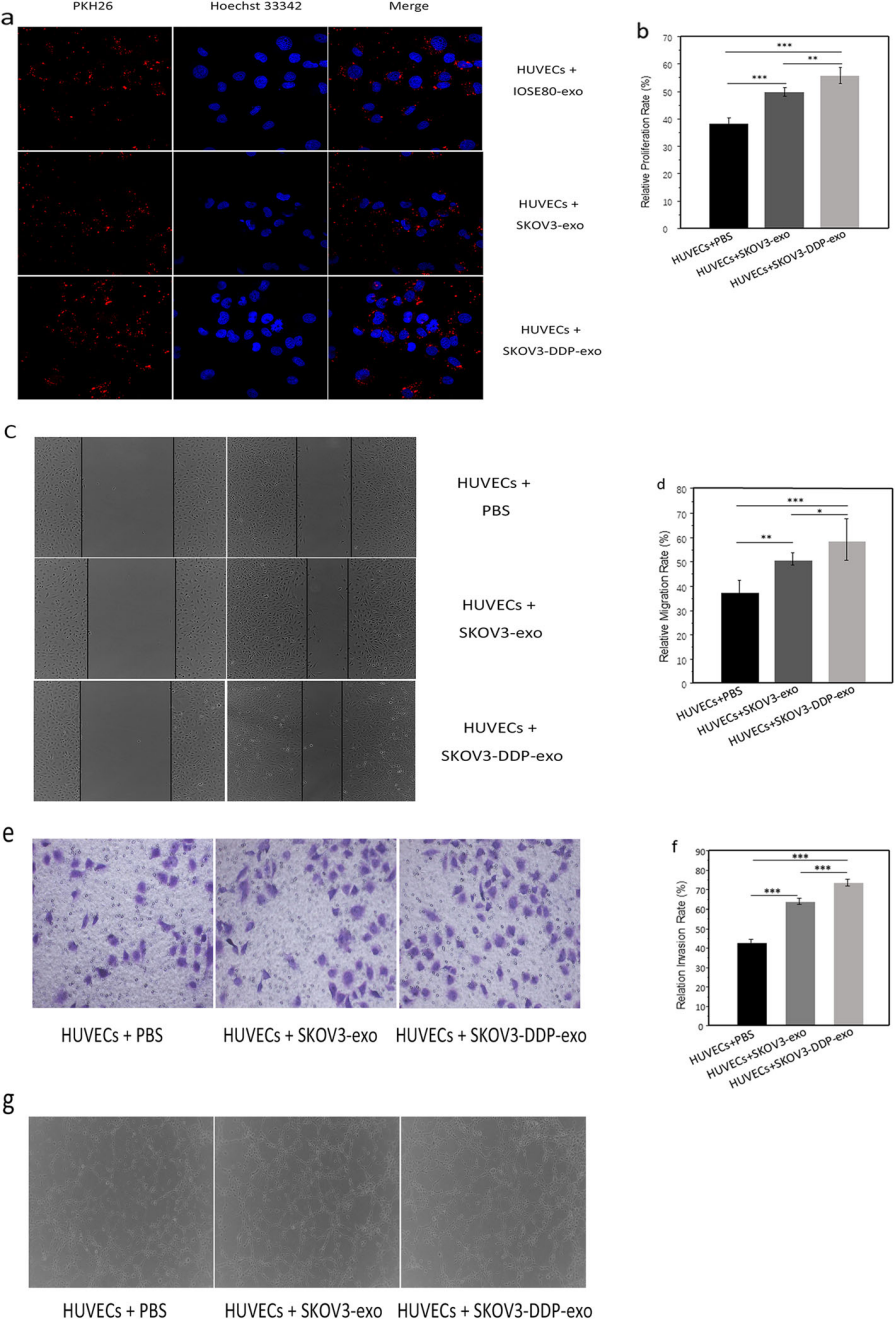
**a** The internalization of IOSE80-exo, SKOV3-exo and SKOV3-DDP-exo (PKH26-labeled, red fluorescence) by HUVECs (Hoechst 33342-labeled, green fluorescence) was examined by confocal laser scanning microscopy. **b** Proliferation of HUVECs co-cultured with SKOV3-exo, or SKOV3-DDP-exo was measured by CCK-8, cells treated with PBS were served as the control. **c**-**d** Migration of HUVECs co-cultured with SKOV3-exo, or SKOV3-DDP-exo was measured by scratch assays. Cells treated with PBS were served as the control. **e**-**f** Invasion of HUVECs co-cultured with SKOV3-exo, or SKOV3-DDP-exo was measured by transwell assay, cells treated with PBS were served as the control. **g** Capillary-like tubes of HUVECs co-cultured with SKOV3-exo, or SKOV3-DDP-exo were measured by tube formation experiment, cells treated with PBS were served as the control. *p < .05, **p < .01, ***p < .001

To investigate the uptake of exosomes, HUVECs labeled with hoechst 33342 are incubated with PKH26-labeled exosomes. After 48 h, exosomes labeled PKH26 are detected in HUVECs by confocal laser scanning microscopy. From Fig. 2a, that exosomes enter HUVECs is observed.

Exosomes can be absorbed in vascular endothelial cells. Next, we will investigate whether drug resistant and sensitive ovarian cancer cells can promote angiogenesis. In order to study the influences of exosomes derived from drug sensitive SKOV-3 and resistant SKOV3-DDP cells on angiogenesis, exosomes are cultured with HUVECs for 48 h. We use CCK-8 assay to evaluate cell viability. As shown in Fig. 2b, SKOV3-exo and SKOV3-DDP-exo promote the growth of HUVECs than PBS treatment group (*p*< 0.001 vs *p*< 0.001), and SKOV3-DDP-exo prompts the proliferation of HUVECs cells more significantly (*p*=0.001). Similarly, SKOV3-exo and SKOV3-DDP-exo promoted the migration of HUVECs, and the effects of exosomes derived from SKOV3-DDP cells are more significant (*p*=0.002 vs *p*< 0.001, *p*< 0.05) (Fig. 2c and d). In addition, the intraluminal capillary-like structure formed by HUVECs was observed, SKOV3-exo and SKOV3-DDP-exo induced tube formation, moreover, the exosomes secreted by SKOV3-DDP cells were more effective (*p*< 0.001 vs *p*< 0.001, *p*< 0.001) (Fig. 2e and f). Moreover, exosomes can conduce HUVECs to form tube structure (Fig. 2g).

### Exo-miR-130a released by SKOV3 cells and SKOV3-DDP cells contributes to angiogenesis

**Fig. 3:**
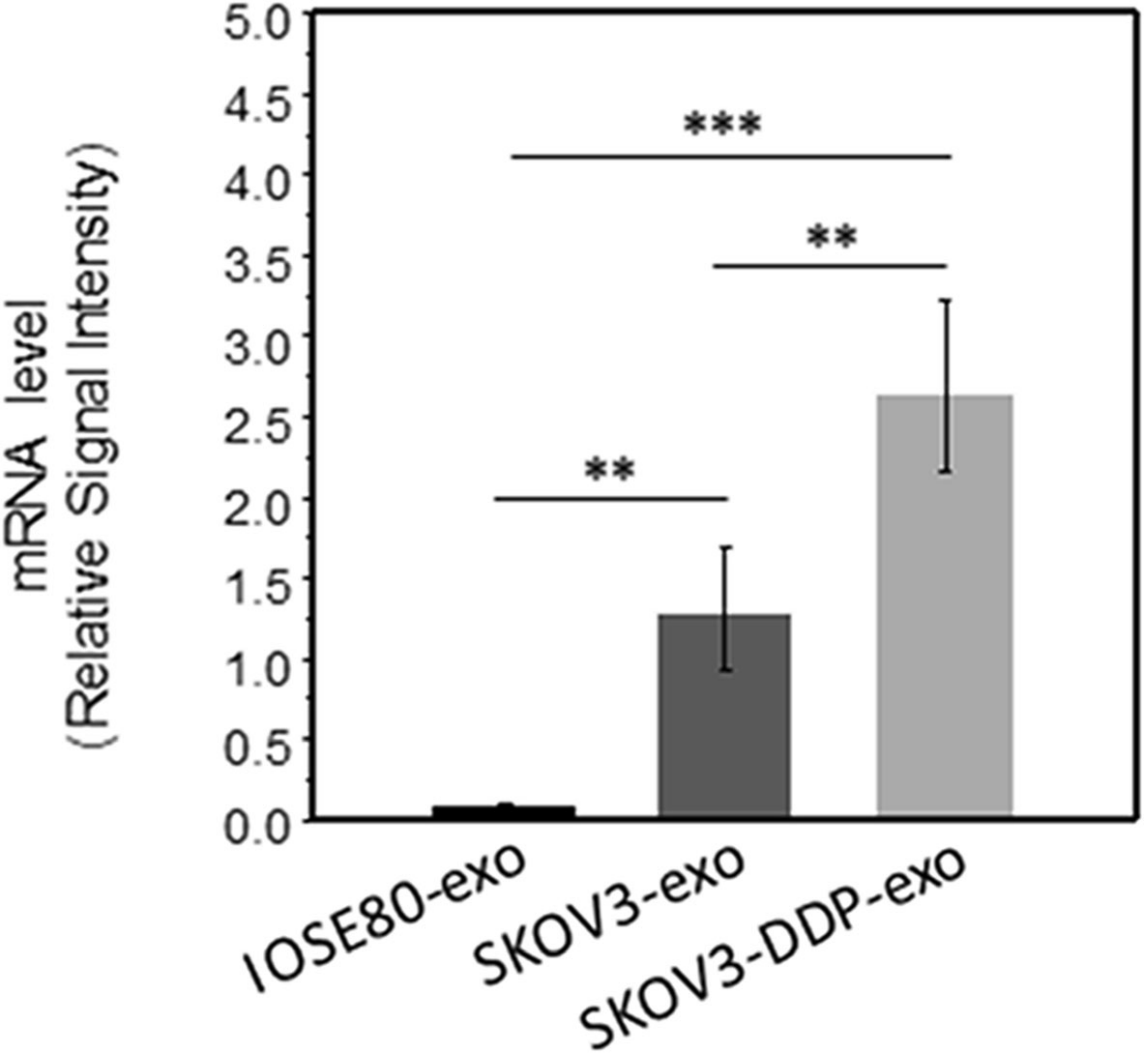
Expression of miR-130a in exosomes derived from SKOV3 and SKOV3-DDP cells

It is generally accepted that the components of exosomes derived from tumor cells can affect the biological behavior of other cells. Our findings show that SKOV3-DDP-exo promote angiogenesis more significantly than SKOV3-exo. We believe that the component of SKOV3-exo is different from SKOV3-DDP-exo, and this component promotes angiogenesis. The role of exosome miRNAs is increasingly prominent. Among them, Haiou found that miR-130a promotes angiogenesis in the development of gastric cancer [24], While miR-130a is found up-expression in ovarian cancer sensitive and resistant cells, and higher expression in resistant cells [25]. Based on the above, we speculate that miR-130a also plays an important role in SKOV3-DDP-exo. By detecting the level of miR-130a, we found that miR-130a expression was significantly higher in SKOV3-DDP-exo (*p*< 0.01 vs *p*< 0.01, *p*< 0.001) (Fig. 3). This is consistent with the study of Johan, the content of exosomes and their biological function depends on the cell of origin [26].

## Discussion

For decades, researchers have revealed that angiogenesis plays a crucial role in tumorigenesis, progression and drug resistance, etc [27, 28] Tumor cells could influence the normal vasculature and stimulate rapid formation of new blood vessels to supply nutrition, however, the mechanism is ambiguous [27]. Recent studies have found that tumor cells can release a variety of bioactive substances to affect the biological behavior of other cells, such as stromal cells and immune cells, thus promoting their own progression, exosomes are involved in tumor progression. Moreover, researchers have found that exosomes secreted by tumor cells can influence angiogenesis. For example, Lu et al. unearth that exosomes secreted by nasopharyngeal carcinoma cells can be absorbed by vascular endothelial cells, in which miR-9 targets MDK of the recipient cells and regulates the PDK/AKT pathway to inhibit angiogenesis, thereby inhibiting the metastasis of nasopharyngeal carcinoma [23]. However, no data shows that exosomes originated from chemoresistant OC cells enhance angiogenesis. In our study, we designed to discover the relationship between SKOV3-DDP cells and endothelial cells. This is the first study to elucidate the communication between chemoresistant OC cells and endothelial cells communication via exosomes. So, we visualized exosomes transfer from SKOV3-DDP to HUVECs. Then, via exosomes, chemoresistant OC cells impacted endothelial cells proliferation, migration and tube formation, namely angiogenesis.

Since researchers found the appearance of chemoresistance, they tried all of the efforts to address chemoresistance. There is strong evidence that miRNAs can be involved in drug resistance. For example, Li et al. have found that miR-9 is lowly expressed in chemoresistant OC cells, which binds to BRCA1, leading to block DNA damage repair and promote the development of drug resistance [25]. exosomes are proven not just excrete waste, but also carry mast molecules, including proteins, mRNAs and miRNA. Through membrane fusion, exosomes enter other cells to influence the biological behavior of other cells. We suspect that miRNA is of great significance in promoting angiogenesis in drug-resistant tumor cells. Besides Haiou et al’s finding [24], Yang et al. also have found that miR-130a rises in OC resistant cells and promotes the expression of P-gp in chemoresistant cells [29]. We detected the level of miR-130a in exosomes derived from SKOV3 and SKOV3-DDP, Our results found that miR-130a is up-regulated in exosomes derived from SKOV3 cells and SKOV3-DDP cells, and it was more significantly expressed in drug-resistant strains, suggesting that miR-130a may play a role in promoting angiogenesis in drug-resistant ovarian cancer cells. These data indicate that exosomes originated from chemoresistant cancer cells can also regulate the angiogenic activities.

In the end, How does the exosome miR-130a secreted by chemoresistant OC cells promote angiogenesis need further investigation, and there is no direct evidence that vascular endothelial cells can promote drug sensitive OC cells insensitively after co-incubated with exosomes secreted by drug-resistant cells. In the future, we will continue to study its specific mechanism in depth.

## Conclusion

Taken together, our data identify exosomes derived from chemoresistant SKOV3-DDP cells prompts angiogenesis. Given the molecular and biological complexity of drug-resistance and angiogenesis, a better understanding of how chemoresistant tumor cells-derived exosomes participate in angiogenesis process represents an important challenge, which can open new paths for the development of novel and effective anti-resistance drugs.

## Data Availability

All data generated and analyzed during this work were included in this article.

## References

[1] Markman M (2013). Current standards of care for chemotherapy of optimally cytoreduced advanced epithelial ovarian cancer. Gynecol Oncol.

[2] Pliarchopoulou K, Pectasides D (2011). Epithelial ovarian cancer: focus on targeted therapy. Crit Rev Oncol Hematol.

[3] Mao L, Li J, Chen WX (2016). Exosomes decrease sensitivity of breast cancer cells to adriamycin by delivering microRNAs. Tumor Biol.

[4] Hanahan D, Weinberg RA (2011). Hallmarks of cancer: the next generation. Cell.

[5] Richards KE, Zeleniak AE, Fishel ML (2016). Cancer-associated fibroblast exosomes regulate survival and proliferation of pancreatic cancer cells. Oncogene.

[6] Aucott SW, Donohue PK, Northington FJ (2004). Increased morbidity in severe early intrauterine growth restriction. J Perinatol: official journal of the California Perinatal Association.

[7] Kapsogeorgou EK, Abu-Helu RF, Moutsopoulos HM (2010). Salivary gland epithelial cell exosomes: a source of autoantigenic ribonucleoproteins. Arthritis Rheum.

[8] Elodie S, Carole N, Bérangère L (2005). ICAM-1 on exosomes from mature dendritic cells is critical for efficient naive T-cell priming. Blood.

[9] Nicolas B, Danielle L, Florence F (2002). TCR activation of human T cells induces the production of exosomes bearing the TCR/CD3/zeta complex. J Immunol.

[10] Knight A (2010). Regulated release of B cell-derived exosomes: do differences in exosome release provide insight into different APC function for B cells and DC?. Eur J Immunol.

[11] Clotilde T, Laurence Z, Sebastian A (2002). Exosomes: composition, biogenesis and function. Nat Rev Immunol.

[12] Tr C, S A, G R (2006). Isolation and characterization of exosomes from cell culture supernatants and biological fluids. Curr Protoc Cell Biol.

[13] Hua F, Declerck YA (2013). Targeting the tumor microenvironment: from understanding pathways to effective clinical trials. Cancer Res.

[14] Nor Eddine S, Agnès N (2013). Targeting the tumor microenvironment for cancer therapy. Clin Chem.

[15] Greening DW, Gopal SK, Xu R (2015). Exosomes and their roles in immune regulation and cancer. Semin Cell Dev Biol.

[16] Costa-Silva B, Aiello NM, Ocean AJ (2015). Pancreatic cancer exosomes initiate pre-metastatic niche formation in the liver. Nat Cell Biol.

[17] Soung YH, Nguyen T, Cao H (2016). Emerging roles of exosomes in cancer invasion and metastasis. BMB Rep.

[18] Yousafzai NA, Wang H, Wang Z (2018). Exosome mediated multidrug resistance in cancer. Am J Cancer Res.

[19] Riches A, Campbell E, Borger E (2014). Regulation of exosome release from mammary epithelial and breast cancer cells – a new regulatory pathway. Eur J Cancer.

[20] Gercel-Taylor C, Atay S, Tullis RH (2012). Nanoparticle analysis of circulating cell-derived vesicles in ovarian cancer patients. Anal Biochem.

[21] Safaei R, Larson BJ, Cheng TC (2005). Abnormal lysosomal trafficking and enhanced exosomal export of cisplatin in drug-resistant human ovarian carcinoma cells. Mol Cancer Ther.

[22] Bai M, Li J, Yang H, et al. miR-135b Delivered by Gastric Tumor Exosomes Inhibits FOXO1 Expression in Endothelial Cells and Promotes Angiogenesis. Mol Ther. 2019;27(10):1772–83. 10.1016/j.ymthe.2019.06.018 PMC6822229 31416776

[23] Lu J, Liu QH, Wang F (2018). Exosomal miR-9 inhibits angiogenesis by targeting MDK and regulating PDK/AKT pathway in nasopharyngeal carcinoma. J Exp Clin Cancer Res.

[24] Yang H, Zhang H, Ge S, et al. Exosome-Derived miR-130a Activates Angiogenesis in Gastric Cancer by Targeting C-MYB in Vascular Endothelial Cells. Mol Ther. 2018;26(10):2466–75. 10.1016/j.ymthe.2018.07.023 PMC6171076 30120059

[25] Li N, Yang L, Wang H (2015). MiR-130a and MiR-374a function as novel regulators of Cisplatin resistance in human ovarian Cancer A2780 cells. PLoS One.

[26] Johan S, Tom W, Sjoerd VR (2008). Glioblastoma microvesicles transport RNA and proteins that promote tumour growth and provide diagnostic biomarkers.

[27] Carmeliet P, Jain RK (2000). Angiogenesis in cancer and other diseases. Nature.

[28] Folkman J (1971). Tumor angiogenesis: therapeutic implications. New Engl J Med.

[29] Yang L, Li N, Wang H (2012). Altered microRNA expression in cisplatin-resistant ovarian cancer cells and upregulation of miR-130a associated with MDR1/P-glycoprotein-mediated drug resistance. Oncol Rep.

